# Mixed adenoneuroendocrine carcinoma of the hepatic bile duct: a case report and review of the literature

**DOI:** 10.1186/s12876-020-01550-2

**Published:** 2020-11-25

**Authors:** Sulai Liu, Zhendong Zhong, Meng Xiao, Yinghui Song, Youye Zhu, Bo Hu, Zengpeng Sun, Weimin Yi, Chuang Peng

**Affiliations:** 1 Department of Hepatobiliary Surgery, Hunan Provincial People’s Hospital/The First Affiliated Hospital of Hunan Normal University, Changsha, Hunan Province People’s Republic of China; 2 Department of Hepatobiliary Surgery, Changsha County People’s Hospital/Hunan Provincial People’s Hospital Xingsha Campus, Changsha, China; 3 Department of Medical Center, Hunan Provincial Hospital for Occupational Diseases, Changsha, China; 4 Department of Pathology, Changsha County People’s Hospital/Hunan Provincial People’s Hospital Xingsha Campus, Changsha, China

**Keywords:** Neuroendocrine tumor, Mixed adenoneuroendocrine carcinoma, Hepatic bile duct

## Abstract

**Background:**

The World Health Organization's updated classification of digestive system neuroendocrine tumors in 2010 first proposed the classification of mixed adenoneuroendocrine carcinoma (MANEC). The incidence of biliary malignant tumors with neuroendocrine tumors accounts for less than 1% of all neuroendocrine tumors. Moreover, the incidence of hilar bile duct with MANEC is very rare.

**Case presentation:**

A 65-year-old female patient came to our hospital for repeated abdominal pain for more than 4 months and skin sclera yellow staining for 1 week. Contrast-enhanced computed tomography imaging and magnetic resonance results suggested a hilar tumor for Bismuth-Corlette Type II. The patient underwent radical surgery for hilar cholangiocarcinoma. Finally, the patient was diagnosed with hilar bile duct MANEC, staged 1 (pT1N0M0) based on the eighth edition of the AJCC. Histopathology showed that the tumor was a biliary tumor with both adenocarcinoma and neuroendocrine carcinoma. No evidence of recurrence and metastasis after 20 months of follow-up.

**Conclusions:**

We first reported a MANEC that originated in the hilar bile duct. As far as we known, there were few reports of biliary MANEC, and the overall prognosis was poor. We also found that the higher the Ki-67 index, the worse the prognosis of this type of patient. Radical surgery is the most effective treatment.

## Background

The most common pathological type of hilar cholangiocarcinoma is adenocarcinoma, accounting for more than 90%. Others mainly include gland-squamous tumors, intraductal papillary tumors, mucinous gland tumors [1]. Mixed adenine neuroendocrine carcinoma (MANEC) is defined as a compound tumor of adenocarcinoma or squamous cell carcinoma mixed with neuroendocrine carcinoma (NEC) or neuroendocrine tumor (NET), with each tumor accounting for at least 30% of the tumor. Meanwhile, these two histological components must be malignant [2]. Neuroendocrine neoplasms (NENs) are distributed in the neuroendocrine system, mainly from the neuroendocrine cells of the digestive system and respiratory tract. The diagnosis depends on histopathological examination [3].NENs can form a mixed carcinoma with malignant-tumors in the primary site, and neuroendocrine tumors of biliary mixed adenocarcinoma are rare in clinical practice [4].

Since the concept of MANEC was introduced in 2010, cases of MANEC have been continuously reported. Retrieving literature, there are only a few case reports describing the MANEC of the hepatic bile duct. However, there is no report of hepatic hilar bile duct MANEC. We presented a patient with hepatic hilar bile duct MANEC here, and combined with the literature to summarize the characteristics of this disease.

## Case presentation

### Case history

A 65-year-old female patient came to our hospital on January 11th, 2018 for repeated pain in the upper abdomen for 4 months and skin sclera for 1 week. At the time of admission, the patient's skin and sclera turned yellow, mild abdominal pain, no fever and abdominal tenderness. Laboratory examination showed obstructive jaundice changes accompanied by an increase in Carbohydrate antigen 19-9 (CA19-9).

**Fig. 1:**
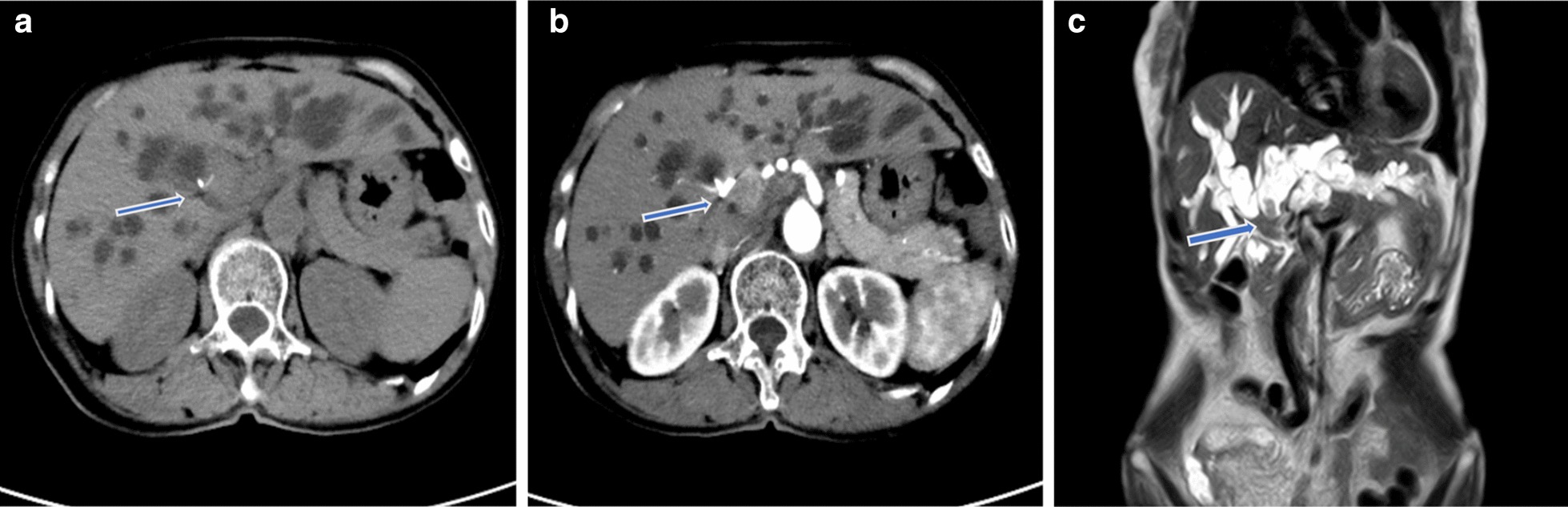
Preoperative imaging findings showed hilar bile duct mass. **a** CT scan showed that the liver tissue area saw soft tissue-like density lesions, and the bile ducts above the lesions were significantly expanded. **b** CT enhanced scan indicates that the lesion is mildly enhanced. **c** MRCP showed truncation of the hilar bile duct, and the intrahepatic bile duct was significantly dilated above the obstruction

The patient underwent a contrast-enhanced computed tomography (CT) and magnetic resonance imaging examination in our hospital. The results showed that soft tissue-like density lesions were seen in the bile duct of the hilar region, and the lesions involved the upper segment of the common bile duct. Enhanced scanning lesions showed mild enhancement. Considering Bismuth-Corlette II type of hilar cholangiocarcinoma, intrahepatic bile duct dilatation was evident above the tumor site. The structure of the portal hepatic hilum area was not clear, considering the possibility of tumor invasion. Multiple lymph nodes were swollen after the retroperitoneum. Magnetic Resonance Cholangiopancreatography (MRCP) showed hepatic hilar bile duct truncation, and the intrahepatic and extrahepatic bile ducts were significantly dilated above the obstruction. No significant expansion of the main pancreatic duct. No intrahepatic or distant metastases were found (Fig. 1).

After detailed preoperative discussion and preparation, resection of the extrahepatic bile ducts with concomitant radical lymphadenectomy and Roux-en-Y cholangiojejunostomy was performed. During the operation, hepatic cholestasis was observed, and the hilar bile duct had a mass of 2 cm × 3 cm × 2 cm. The portal vein has not been invaded by the tumor. No perioperative complications occurred and the patient was discharged on the 12th day after surgery. According to the eighth edition of the AJCC tumor staging [5], this patient was stage 1 (pT1N0M0). After she discharged from hospital, she was followed-up by telephone once a month, and she came to hospital for liver function test, CA19-9 and abdomen contrast-enhanced computed tomography. Until now, no abdominal pain, no skin or scleral yellowing and other abnormalities, no signs of recurrence and metastasis.

### Histology and immunohistochemistry

A 2 × 1.8 × 1 cm mass was seen in the hilar bile duct and invaded the entire bile duct wall. At light microscope, two tumor components of adenocarcinoma and neuroendocrine cancer could be seen as collision type. Adenocarcinoma cells were columnar, cubic, and nuclear division were rare. Neuroendocrine cancer tumor tissues were solid, flaky, trabecular or organ-like. The cells were small and round, and the cytoplasm were sparse. There were abundant sinusoids in the middle, and the Ki-67 index was 70%. Neurological invasion was occurred, no intravascular tumor thrombus was seen, and no involvement of cancer in the bile duct margin. No lymph node metastasis.

**Fig. 2:**
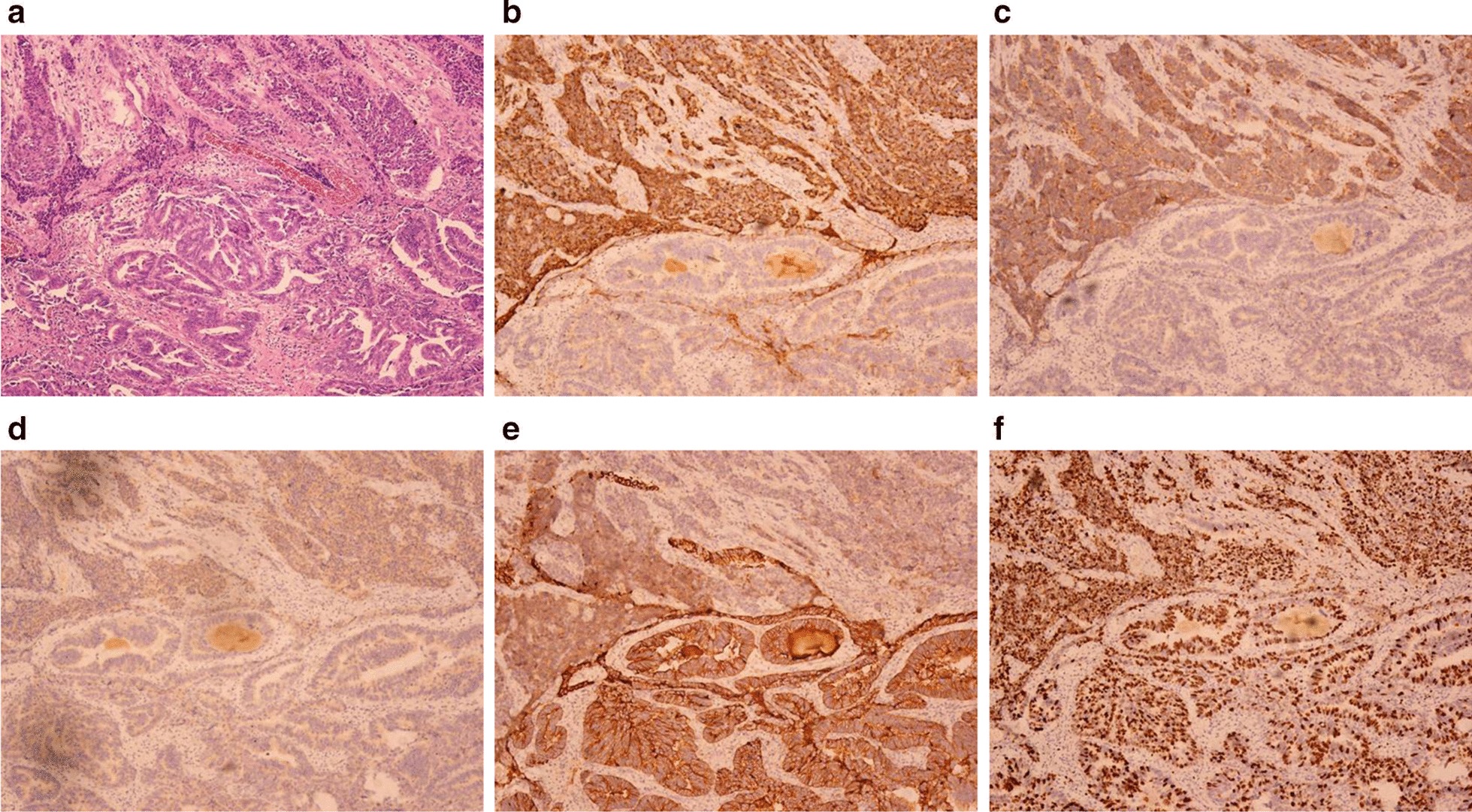
Histology and immunohistochemistry. **a**Neuroendocrine carcinoma and tubular adenocarcinoma [hematoxylin and eosin staining (HE), ×100]. **b** Cytoplasmic diffuse positive in CgA cells (DAB color development, ×200). Immunohistochemical staining. **c** Syncytoplasmic cytoplasmic positive, ×200. **d** Neuronal specific enolase (NSE) positive in neuroendocrine tumor cells, ×100. **e** Cytoplasmic cytokeratin 7 (CK7) diffuse positive in adenocarcinoma tumor cells, ×200. **f** Cell proliferation index (Ki-67) cell nucleus positive, positive rate was 70%, ×100

Immunohistochemistry and special staining: CK7 cholangiocarcinoma ( +), CK19 ( +), CD34 vascular ( +), NSE nerve ( +), Ki67 (+ , 70%), CgA, Syn neuroendocrine carcinoma ( +), p53 (cholangiocarcinoma 3 + , neuroendocrine carcinoma 2 +), VG ( +) (Fig. 2).

## Discussion and conclusion

90% of the malignant tumors of the biliary system are adenocarcinomas, and other types of tumors are rare [6]. The proportion of biliary NENs to all NENs is less than 1%. MANEC, which originated in the hilar bile duct, is rare. The World Health Organization's 2010 classification system classifies neuroendocrine tumors into neuroendocrine tumors (NET), neuroendocrine carcinomas (NEC), and MANEC. Subdivided into NET G1 phase: carcinoid, < 2 mitotic cells per 10 high power fields, and/or Ki-67 index ≤ 2%. NET G2 phase: 2–20 mitotic cells per 10 high power fields, and/or Ki-67 index between 3 and 20%. NET G3: Neuroendocrine carcinoma with > 20 mitotic cells per 10 high power fields, and/or Ki-67 index > 20%, and MANEC [7, 8].

According to the location of tumor components, MANEC can be divided into three subtypes: composite neoplasms, collision neoplasms and combined neoplasms. Composite neoplasms refers to two different types of tumors separated from each other with normal tissue separation in between. Collision neoplasms are the neuroendocrine and exocrine components occur in separate areas of the same lesion with no normal tissue spacing in between. While in other MANECs they are intimately and diffusely admixed (combined neoplasms) [9]. In this case, the patient's tumor was located in the hilar bile duct, and no intrahepatic bile duct was accumulated. The two tumor components were separated from each other, which was a collision type. The proportion of the two tumor components was greater than 30%.

By searching the literature, we found that biliary MANEC is rare, and only one of the retrospective studies mentioned two cases of hepatic MANEC. But it lacked corresponding analysis [10] 0.10 cases of biliary MANEC patients were summarized [10–18]. Including 4 cases of distal bile duct, 2 cases of hilar bile duct, 1 case of common bile duct, 1 case of intrahepatic bile duct, 1 case of cystic duct, and 1 case of extrahepatic bile duct. The symptoms of this type of patients mainly include abdominal pain and yellowing of the skin or sclera. CT/magnetic resonance imaging is consistent with the characteristics of biliary adenocarcinoma. Accurate diagnosis depends on histopathological examination. Preoperative tumor markers were generally not elevated. All patients underwent radical surgery. The type of tumor was mainly mixed with adenocarcinoma and neuroendocrine carcinoma. Only two cases were considered as collision type MANEC. At present, the pathogenesis of bile duct MANEC is unclear. Some scholars believe that it may be related to the neuroendocrine of a small amount of enterochromaffin cells distributed on the biliary system [19], and some are believed to be caused by congenital developmental abnormalities or long-term chronic inflammation. Regarding the pathogenesis of MANEC, Harada et al. [20] analyzed the histopathological features of 274 cases of biliary neuroendocrine tumors, suggesting that hepatic stem cells may be involved in the differentiation of neuroendocrine tumor cells. It is also suggested that bile duct stones combined with cholangitis is one of the important risk factors that may induce the accidental generation of neuroendocrine cells. Some scholars have also suggested that the Notch1-Hes1 signal axis inhibits the differentiation of neuroendocrine cells and maintains the tubular or acinar characteristics of adenocarcinoma and normal biliary cells. The interruption of this signal axis may be related to the occurrence of biliary MANEC [21].

All MANEC are basically diagnosed by histopathological examination [15]. It is reported that cytological biopsies by ERCP or endoscopic ultrasound-guided fine needle puncture etc. were used before surgery. But only AC or NET was detected, and the postoperative diagnosis was MANEC [22]. The possible reason may be the diverse components of MANEC, and the complicated structural distribution of AC and NEC leading to incomplete sampling. Therefore, it is still necessary to explore other effective examination methods to assist the preoperative medical examination to make a diagnosis of the bile duct MANEC. As a rich blood supply tumor, NET is obviously strengthened in the CT phase of the arterial phase, but the cholangiocarcinoma is poorly strengthened. It is reported that MANEC with obvious enhancement in the CT arterial phase, which proves the existence of the NET component and assists before surgery, which supported a new direction for the diagnosis of bile ducts MANEC [15]. Preoperative lymph node biopsy can also be used as an auxiliary diagnostic method. When the metastatic component is NETG2 or NEC, the possibility of MANEC should be considered [10].

Because the MANEC of the biliary tract is rare, the clinical research lacks sufficient data, and the existing case reports indicate that the prognosis is poor. There is literature analysis that the tumor-free survival and overall survival of the biliary neuroendocrine tumor stage are 5.8 (range 0.4–53.6) months, and 13.7 (range 0.7–102.1) months, respectively. And the prognosis of NET is significantly better than NEC and MANEC. The higher the Ki67 index, the easier the tumor recurs and the poor prognosis [23]. It has been reported in the literature that adjuvant radiotherapy and chemotherapy after neuroendocrine tumors may improve the prognosis, but radical surgical resection is still the most effective treatment [10, 21]. This patient also underwent radical surgery. For hilar cholangiocarcinoma treatment, our central has extensive treatment experience [24]. She still survived very well after surgery and did not receive any radiotherapy or chemotherapy and other adjuvant treatment.

At present, most of the evidence indicates that the treatment of biliary tract MANEC should be based on the grade of adenocarcinoma or NET, and the high grade tumor as a reference for postoperative adjuvant therapy. Multiple retrospective analyses have pointed out that the Ki-67 index is an independent risk factor for predicting the prognosis of neuroendocrine carcinoma. When the Ki-67 index is higher than 55%, it is highly prognostic [8, 25, 26].

In conclusion, the overall survival of biliary MANEC is still lacking in large sample statistics. We reported the first case of MANEC in the hilar bile duct. It is hoped that reference materials will be provided for the diagnosis, treatment and prognosis analysis of such patients.

## Data Availability

Data and materials are included in the manuscript.

## Abbreviations

ADC: Adenocarcinoma
CA19-9: Carbohydrate antigen 19-9
CBD: Common bile duct
CK7: Cytoplasmic cytokeratin 7
CT: Computed tomography
HE: Hematoxylin and eosin staining
IPNB: Intraductal papillary neoplasm of the bile duct
MANEC: Mixed adenoneuroendocrine carcinoma
MRCP: Magnetic resonance cholangiopancreatography
NEC: Neuroendocrine carcinoma
NENs: Neuroendocrine neoplasms
NET: Neuroendocrine tumor
NSE: Neuronal specific enolase
PD: Pancreaticoduodenectomy
UICC: Union for international cancer control
